# Effects of Extra Virgin Olive Oil (EVOO) and the Traditional Brazilian Diet on Sarcopenia in Severe Obesity: A Randomized Clinical Trial

**DOI:** 10.3390/nu12051498

**Published:** 2020-05-21

**Authors:** Erika Aparecida Silveira, Jacqueline Danésio de Souza, Ana Paula dos Santos Rodrigues, Ricardo M. Lima, Camila Kellen de Souza Cardoso, Cesar de Oliveira

**Affiliations:** 1 Postgraduate Program in Health Sciences, Faculty of Medicine, Federal University of Goias, Goiânia 74605-220, Goias, Brazil; jackdanesio@yahoo.com.br (J.D.d.S.); anapsr@gmail.com (A.P.d.S.R.); 2 Affiliate Academic at the Department of Epidemiology & Public Health, Institute of Epidemiology & Health Care, University College London, London WC1E 6BT, UK; 3 Faculty of Physical Education, University of Brasília, Darcy Ribeiro University Campus, Brasília CEP 70910-900, Distrito Federal, Brazil; ricardomoreno@unb.br; 4 School of Social Sciences and Health, Nutrition Course, Pontifical Catholic University of Goias, Goiânia 74605-020, Brazil; camilacardoso_nut@hotmail.com; 5 Department of Epidemiology & Public Health, University College London, London WC1E 6BT, UK; c.oliveira@ucl.ac.uk

**Keywords:** diet, sarcopenia, grip strength, walking speed, body composition, obesity, clinical trial

## Abstract

Background: Nutritional interventions may have positive effects on sarcopenia and body composition. Objective: to evaluate the effectiveness of extra virgin olive oil (EVOO) consumption and a healthy traditional Brazilian diet (DieTBra) on improving sarcopenia indicators and reducing total body fat in severe obesity. Methods: A randomized controlled trial registered at ClinicalTrials.gov (NCT02463435) conducted with 111 severely obese participants randomized into three treatment groups—(1) EVOO (52 mL/day), (2) DieTBra, (3) DieTBra + EVOO (52 mL/day)—for 12 weeks. Body composition was assessed by dual-energy X-ray absorptiometry and sarcopenia by walking speed and handgrip strength. Results: Significant reductions in total body fat (*p* = 0.041) and body weight (*p* = 0.003) were observed in the DieTBra group. In the DietBra + olive oil group there was also a significant reduction in body weight (0.001) compared to the olive oil-only group. ANCOVA analyses showed reductions in total body fat in the DieTBra (*p* = 0.016) and DieTBra + olive oil (*p* = 0.004) groups. Individuals in the DieTBra group had significant improvements in their walking speed (*p* = 0.042) and handgrip strength (*p* = 0.044). Conclusions: DieTBra contributes to improvements in handgrip strength, walking speed, and total body fat in severely obese adults. The major study was registered at ClinicalTrials.gov (NCT02463435).

## 1. Introduction

Obesity is a chronic non-communicable disease (NCD) with complex and multifactorial etiology, mainly characterized by the presence of excess adipose tissue [1]. Severely obesity (i.e., body mass index (BMI) ≥ 35 kg/m^2^) affects approximately 2.3% of men and 5.0% of women worldwide and has exhibited the highest growth rate among the stages of obesity in recent years [2]. Thus, obesity is a relevant public health problem that is directly related to increased mortality, decreased life expectancy, and increased years of life lost [3]. Strategies to improve morphological and functional status and quality of life in these individuals are clearly needed.

Fat mass increases in obesity can occur concomitantly with a decrease in muscle mass, and this combination has been referred to as sarcopenic obesity [4]. Sarcopenic obesity has been associated with decreased muscle strength and physical function, more than obesity or sarcopenia alone, and has been recently examined as an important cause of frailty [5,6,7]. Sarcopenic obesity has been also associated with an increased risks of functional disability, falls, fractures, cardiovascular diseases, and metabolic disorders [6,8,9,10,11,12]. The pathogenesis of sarcopenic obesity is multifactorial, encompassing neurological, hormonal, environmental, genetic, and nutritional factors, and their interplay [13,14].

Nutritional interventions and other non-pharmacological therapies should be the first choice of treatment for several NCDs, in cases where the participant is not at imminent risk of death [3,15], including obesity. Observational studies have demonstrated a positive effect of the Brazilian food pattern on the incidence of NCDs, such as cardiovascular disease, hypertension, and diabetes [16,17]. Therefore, the traditional Brazilian diet (DieTBra), characterized by a healthy dietary pattern with low consumption of processed and ultra-processed foods, may serve as a good option for the treatment of sarcopenic obesity-related phenotypes. Moreover, recent reports have provided evidence that regular consumption of extra virgin olive oil (EVOO) has positive effects on body composition, including improvements in muscle tissue structure and function [18,19,20,21]. DieTBra and the consumption of EVOO can thus be potential alternatives for increasing lean mass, decreasing body fat, and improving muscle strength and functionality [4,18,20,21]. However, to our knowledge, no studies have investigated the effect of these interventions on phenotypes related to obesity and sarcopenia.

It has been well documented that inappropriate dietary intake and energy density of the diet are among the major contributing factors to the increase of obesity globally. It has also been recognized that improving diet status over the lifespan is an important strategy to preventing sarcopenia in older age [22]. Given the high impacts of nutrition on the obesity epidemic and sarcopenia development [22,23], it is important to analyze the effectiveness of dietary interventions as therapeutic and/or preventive measures against the aggravation of these health problems. Therefore, the aim of the present study was to assess the effectiveness of extra virgin olive oil consumption and a healthy dietary pattern (DieTBra) on improving sarcopenia indicators and reducing total body fat in adults with severe obesity.

## 2. Materials and Methods

### 2.1. Study Design

The present randomized controlled trial was conducted between June 2015 and February 2016, in Goias (Brazil). This trial was on free-living subjects and was part of the “Effect of nutritional intervention and olive oil in severely obesity (DieTBra Trial)” project registered at clinicaltrials.gov (NCT02463435) on 4 June 2015. Details of the study design, subject recruitment, and randomization were previously described [24,25,26,27].

### 2.2. Participants

This study included 111 participants with BMI ≥ 35 kg/m^2^ [1,2], aged between 18 and 64 [28], who were recruited from the primary care service of the Brazilian Unified Health System, Goiânia, Goias State, in Central Brazil. Participants were referred to the nutrition in severe obesity out participant clinic, and the study took place at the Clinical Research Unit of the Clinical Hospital/Federal University of Goias. Participants were residents in Goiânia or in its metropolitan region. Those who had previously undergone bariatric surgery, were pregnant and/or lactating, had a physical and/or mental deficiency, had exhibited an >8% reduction in weight over the least 3 months, received nutritional or medical supervision for weight reduction, received some type of nutritional treatment in the last 2 years, were using anti-obesity drugs, or had a food allergy to some type of vegetable oil were excluded from the present study. Participants who did not meet the specifications for performing dual energy X-ray absorptiometry (DXA), i.e., body weight ≥ 130 kg, pacemakers, or metallic implants, were also excluded.

The sample size calculation was performed based on the central limit theory, since the parameters in the scientific literature for the calculation were not observed at the moment of the study design [29]. Thus, a number of 50 participants was estimated for each intervention arm, and a total of 150 for the randomization process.

### 2.3. Randomization, Blindness, and Quality Control

**Figure 1:**
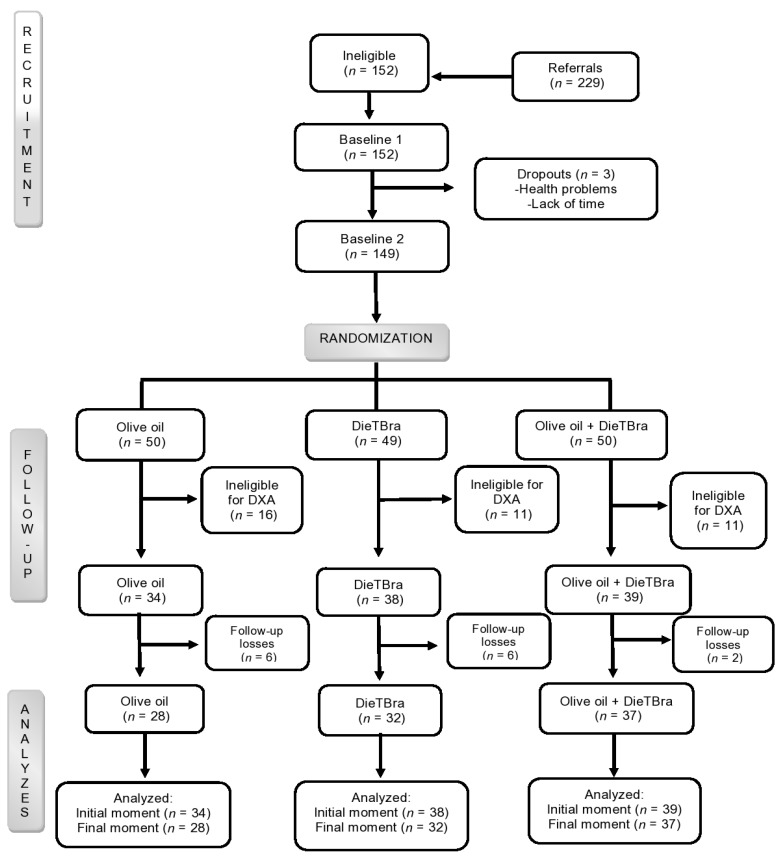
Flowchart of the severely obese participants in the study.

Participants were randomized into three treatment groups, with a 1:1:1 allocation and parallel intervention. Randomization was performed using an algorithm available at www.randomization.com. The allocation of participants was performed by the same trained researcher in a separated room. The intervention groups were: (1) extra virgin olive oil (EVOO), of which participants received 52 mL/day; (2) traditional Brazilian diet (DieTBra); and (3) DieTBra + olive oil, which received the same intervention as the DieTBra group in addition to EVOO supplementation of 52 mL/day. The participant flow in the study is shown in Figure 1.

All participants were blinded to the type of oil used. To ensure blindness, the intervention groups were examined on different days, thereby avoiding contact between participants. Additionally, throughout the study, the term “polyphenol-rich food supplement” was used instead of “extra virgin olive oil” to avoid potential bias. The sachet of extra virgin olive oil delivered to the participants was packaged without any label in order to ensure non-identification of the product.

The quality of the collected data was guaranteed by training the research team members regarding the protocols of care and intervention, the approaches on how to minimize loss to follow-up, and the routine service. The intervention was conducted over 12 weeks, with monthly follow-up visits.

### 2.4. DieTBra Intervention

The DieTBra group was subjected to a dietary intervention based on the Food Guide for the Brazilian Population, which comprises a balanced diet, fractioned in 4–6 meals/day, and an individualized food plan calculation [15,17,30,31,32]. The DieTBra rescues the Brazilian healthy eating habits used before the nutritional transition, which consisted of consumption of fresh and minimally processed foods, and the non-ingestion of industrialized foods and beverages classified as ultra-processed [15]. Main meals (lunch and dinner) are based on the consumption of rice, beans, a small portion of lean meat, and raw and cooked vegetables. Fresh fruits, bread, milk, and dairy are consumed in small meals. This dietary prescription was culturally acceptable, economically accessible, fresh and minimally processed, and had adequate amounts of fruits and foods rich in antioxidants [17,31,32].

The macronutrient distribution for the DieTBra group was set according to the Dietary Reference Intakes (DRIs) from the Institute of Medicine [33], those being 45%–65% carbohydrates, 10%–35% proteins, and 20%–35% lipids. In relation to lipid consumption, the Brazilian Society of Cardiology recommends <20% saturated fats, 55% mono-unsaturated, and 25% polyunsaturated fats [34]. The prescribed diet was balanced according to the recommendations of healthy eating, distributed in 5 or 6 meals per day. Throughout the follow-up, the effectiveness of the intervention was evaluated through the analysis of dietetic data on the dietary intake of DieTBra, in order to guarantee the fulfillment of the prescribed diet.

To estimate the total energy value (TEV) of the food plan, we considered the energy expenditure at rest (EER) using the following equation developed for severely obese individuals: EER = 560.43 + (5.39 × W) + (14.14 × FFM), where W is the current weight in kg, and FFM, the fat-free mass value evaluated by multi-frequency electric bioimpedance (Inbody S10^®^) [35]. The total energy expenditure (TEE) was calculated by multiplying EER by the activity factor (AF) and the thermic effect of food (TEF) [35]. The AF was determined based on information collected from the Global Physical Activity Questionnaire (GPAQ), while considering the TEF as 8% of the TEE [36,37,38]. The participants received an individualized food plan, which aimed to reduce the initial body weight by 5%–10% according to the BMI range.

After calculating the desired weight loss percentage, the TEV was determined using a daily caloric reduction (550–1100 kcal/day) according to the individual goal of weekly weight reduction (0.5–1.0 kg/week) [39]. The macronutrient distribution of TEV followed the dietary reference intake (DRI) [33].

### 2.5. EVOO and DieTBra + EVOO Interventions

Participants in the olive oil group were instructed to consume 52 mL/day of EVOO. In this group, the participants received only nutritional supplementation with EVOO, and no other type of food intervention was performed. In the DieTBra + EVOO group, dietary intervention was performed as described previously, along with supplementation of 52 mL of EVOO per day (four sachets, including two at lunch and two at dinner, totaling 468 kcal/day). Although this figure was discounted in the quantities of prescribed foods, this group still maintained a hyperlipidic dietary prescription (approximately 45%).

For the groups that received olive oil, a package with individual sachets (13 mL each) was delivered at the end of each visit for monthly consumption. The amount of olive oil was determined based on studies that found beneficial effects with dosages between 25 and 53 mL/day [39]. It should be noted that the planned quantity was on average 42% higher than commonly found in scientific literature (≤30 mL/day), because we considered the losses during ingestion [39,40,41,42]. The consumption losses of the extra virgin olive oil were assessed by a questionnaire developed to evaluate compliance and by collecting and counting the empty sachets returned in the consultations with the nutritionist.

### 2.6. Anthropometry and Body Composition

Weight was measured in kilograms using a duly calibrated digital weighing scale (WELMY^®^), with a capacity of 200 kg and precision of 100 g. Height was determined using a stadiometer, coupled to the weighing scale, with an accuracy of 0.1 cm [43]. The calculation and classification of BMI followed the WHO recommendations [1].

Body composition was assessed by dual-energy X-ray absorptiometry (DXA) using the GE Healthcare model (Lunar DPX NT, Auckland, New Zealand) before and after the intervention period, with a capacity of 130 kg and a width of 1.03 m. In brief, subjects laid face up on the DXA table with their bodies carefully centered. For 47.2% of participants who were larger than the DXA scanner table (>1.03 m), the half-scanner protocol (Hemiscan) was used. These participants were placed in the center of the bed line so that the right side of the body was completely included in the scanner field, and the total composition was estimated by automatic duplication of the right side [44]. This exam was always performed by the same technician from the Clinical and Sports Nutrition Research Laboratory of the Faculty of Nutrition (LABINCE-FANUT) of the Federal University of Goias, in the presence of one of the authors.

The appendicular skeletal muscle mass (ASMM) was determined from the sum of the no osseous lean mass of the arms and legs. The total ASMM was recorded, along with the ASMM adjusted by the height squared (appendicular skeletal muscle mass index (ASMMI)) and the ASMM divided by the BMI (ASMM/BMI) [45,46,47,48]. The total body fat and body fat percentages were assessed by the DXA.

### 2.7. Sarcopenia-Muscle Strength and Gait Speed Test

Handgrip strength (HGS) was used as a measure of muscle strength and was assessed using a JAMAR^®^ hydraulic hand dynamometer. Evaluations were performed in three consecutive trials at 1 min intervals, with the dominant upper limb in the orthostatic position and extended close to the body. The highest value among the attempts was considered for subsequent analyses, and was expressed in kilograms of strength [49,50].

The gait speed test was performed in a flat and unobstructed 6 m corridor; the course was completed three times at the usual speed with an average interval of one minute between each evaluation. The time required to travel the route was recorded by a digital timer, and the average value of the measurements obtained was used for classification [51,52].

### 2.8. Sociodemographic and Lifestyle Characteristics

Sociodemographic characteristics evaluated included sex, age, skin color, socioeconomic class, marital status, and completed years of schooling. The socioeconomic class was evaluated according to the Economic Classification Criteria of the Brazilian Association of Research Companies [53]. With regard to smoking, the participant was classified as non-smoker, smoker, or ex-smoker [54]. 

Alcohol consumption was verified using the Gender, Alcohol and Culture: An International Study (GENACIS) questionnaire [55]. Alcohol consumption was considered excessive when there was ingestion of ≥5 doses of alcoholic beverage for a man and ≥4 doses for a woman on a single occasion; a dose of alcoholic beverage was standardized as 13 g of ethanol [55].

To evaluate physical activity, a triaxial accelerometer (ActiGraph wGT3X) was used. The equipment was worn by the participants 24 h a day for 6 consecutive days. The average value obtained per day was multiplied by seven (weekly value) to determine the average number of minutes of physical activity performed [56]. The intensity of physical activity was classified according to the World Health Organization (WHO) criteria [57]. The mean minutes per day spent on sedentary behavior was also evaluated.

### 2.9. Statistical Analysis

Data were included in a database via double entry with EPI DATA^®^ version 3.1 for consistency and validation analysis. The analyses were performed using STATA/SE software, version 12.0. Data distribution was assessed using the Kolmogorov–Smirnov test. Descriptive analysis was performed according to frequencies, means, and standard deviations.

The primary endpoint of the study included the ASMMI [ASMM/height^2^], whereas the secondary ones included HGS, gait speed, ASMM/BMI, total ASMM, total body fat, and body fat percentage. The outcomes were evaluated continuously, and delta change values were calculated.

To analyze data at baseline across the three groups, Fisher’s exact test or Pearson’s chi-square test and analysis of variance (ANOVA) were performed. The intention-to-treat analysis considered all the subjects included in the clinical trial using the unpaired Student’s *t*-test. The difference between the initial, final, and mean intergroup differences was examined using Student’s *t*-test and ANOVA. The presence of outliers was analyzed using boxplot graphs for all variables.

The analysis of covariance (ANCOVA) was used to analyze the effects of the covariates delta weight, delta physical activity, delta time of sedentary behavior, sex, and age on the outcomes. ANCOVA was performed using variables that met the test requirements, including normal distribution, a linear relationship between the outcome variables and covariates, and homoscedasticity of variances [58]. 

The effect size of the outcomes was calculated for those with *p* values < 0.05 using the Cohen/lbecker test.

### 2.10. Ethical Aspects

The study was conducted in accordance with the Declaration of Helsinki for experiments with human beings. The subjects who participated in the study voluntarily signed an informed consent form prior to participation. The study was approved by the Research Ethics Committee of the Clinical Hospital of the Federal University of Goias (protocol number 747.792).

## 3. Results

Figure 1 presents the flowchart of participants from recruitment to study completion. In brief, a total of 229 participants were assessed for eligibility, of whom 149 met the inclusion criteria. Next, 38 participants were excluded for not meeting the criteria for performing the DXA assessment. After the exclusion criteria were applied, a total of 111 participants underwent all baseline assessments and the level of compliance of the study was 87.4%. The mean intake of extra virgin olive oil was 38.1 ± 11.1 mL/day. In the olive oil group it was 38.8 ± 10.5 mL/day, and in the DieTBra + olive oil group it was 37.5 ± 11.7 mL/day; there were no statistically significant differences between groups. 

There were no significant differences between the groups in terms of their baseline characteristics and outcome variables (*p* > 0.05). Mean BMI value at baseline was 43.7 ± 4.5 kg/m^2^; 68.5% had BMI values between 40 and 49 kg/m^2^. Mean ASMMI and body fat percentage were 8.2 ± 1.3 kg/m^2^ and 51.7% ± 5.1%, respectively (Table 1).

The mean caloric intakes at baseline, during intervention, and post-intervention for the three groups were as follows: olive oil group 1626.0 ± 678.2 Kcal/day, 1496.5 ± 615.4 Kcal/day, and 1464.3 ± 857.3 Kcal/day; DieTBra group 1702.5 ± 597.8 Kcal/day, 1318.8 ± 482.3 Kcal/day, and 1053.7 ± 580.5 Kcal/day; DieTBra and olive oil group 1830.6 ± 1097.3 Kcal/day, 1344.7 ± 513.8 Kcal/day, and 1296.3 ± 512.4 Kcal/day. No differences for caloric intake were observed at baseline and during the intervention between groups (*p* > 0.005). There were no statistical differences between the groups at baseline in macronutrient data for carbohydrates (%; *p* = 0.574), proteins (%; *p* = 0.268), and total fats (%; 0.489). The average protein percentages were 15.69 ± 4.09 in the olive oil group, 17.42 ± 4.65 in the DieTBra group, and 18.19 ± 5.02 in the DieTBra and olive oil group.

In the intention to treat analyses, when baseline and final values were compared in each group, no significant differences were observed (Table 2). Similarly, no significant differences in the post-intervention stage were observed regarding outcomes mean values either between the three groups or when each of intervention was compared another intervention; i.e., paired comparisons (Table 3).

In the comparison of outcome deltas between the three intervention groups, a significant difference was observed for weight (*p* = 0.002). Subsequent paired comparisons revealed a significant difference in total body fat when comparing the olive oil group and the DieTBra + olive oil group (*p* = 0.026), one in body weight between the olive oil group and the DieTBra group (*p* = 0.003), and one between the olive oil group and the DieTBra + olive oil group (*p* = 0.001) (Table 4).

The covariate delta weight met the requirements for the ANCOVA test in the DieTBra and DieTBra + olive oil groups. The delta weight affected the outcomes decreasing the delta total body fat in the DieTBra (*p* = 0.016) and DieTBra + olive oil (*p* = 0.004) groups, and increasing the delta walking speed (*p* = 0.042) and delta HGS (*p* = 0.044) in the DieTBra group. The delta sedentary behavior also met the requirements for ANCOVA in the DieTBra + olive oil group; in particular, the delta sedentary behavior influenced the reduction in total body fat in the DieTBra + olive oil group (*p* = 0.001) (Table 5).

When calculating the effect sizes for the outcomes, we found that the effect of the intervention was considered small (<0.30) for the evaluated outcomes that presented *p* values < 0.05.

## 4. Discussion

To the best of our knowledge, this is the first randomized clinical trial to examine the effects of DieTBra and EVOO consumption on sarcopenia traits in adults with severe obesity. In fact, only a few reports have been conducted on the effects of dietary interventions on parameters of sarcopenia and obesity [4,18]. Therefore, this study makes an important contribution to the scientific knowledge in this area. Our main findings showed that a nutritional diet intervention, with or without extra virgin olive oil supplementation, was effective at improving handgrip strength and walking speed and in reducing body fat in adults with class II/III Obesity. These results provide some evidence supporting that DieTBra could help the treatment of severe obesity and improve sarcopenic indicators. It is well known that body fat reduction and improvements in strength and muscle functionality are the main challenges in obesity treatment [5,10]. Our study demonstrated that these challenges could be overcome using a nutritional intervention.

The interventions investigated had a positive effect on the reduction in total body fat in the DieTBra and DieTBra + olive oil groups. A healthy dietary pattern can improve anthropometric measurements [27,59]. However, there is not enough evidence on nutritional interventions regarding EVOO and a healthy dietary pattern in terms of sarcopenic-related outcomes and other body composition variables in class II/III obesity [27,60]. It is a relevant result because the presence of excessive adipose tissue promotes the storage of circulating lipids at ectopic sites, and skeletal muscle tissue is a potential target site in this process [61,62]. The deposition of fatty acids around and within muscle mass impairs the functionality and quality of muscle, and its capacity to generate force in relation to the muscle area [62]. In the sarcopenic and obese population, high body adiposity is considered an independent predictor of functional limitation and physical disability [61,63,64].

The DieTBra group also had a positive effect on increases in walking speed and handgrip strength. A recent study with obese, sarcopenic, and non-sarcopenic women showed that a short-term weight loss program combining calorie restriction and aerobic exercise can significantly reduce fat mass [18]. In parallel, a European study in elderly women indicated a positive relationship between greater adherence to a Mediterranean dietary pattern, which is also a healthy dietary pattern like DietBra, and higher percentages of fat-free mass, muscle mass, and manual grip strength, related to age [20]. These findings demonstrate the importance of diet therapy through a healthy, affordable, and low-cost diet [65] to improve the indicators of functionality and strength in severely obese individuals. 

The combination of a healthy eating pattern such as DieTBra with regular consumption of EVOO can potentially improve the structure and function of muscle tissue, thereby increasing protein metabolism, redox balance, mitochondrial biogenesis, and anti-inflammatory capacity [19]. It is also worth noting that a dietary intervention based on healthy eating and different aspects of alimentary behavior should be encouraged, which can yield better results in terms of increase in muscle mass and decrease in body fat [30,32]. The addition of EVOO to the DieTBra, however, did not promote further improvements on the studied muscle-related traits. It has been suggested that oxidative stress and low-grade chronic inflammation partially explain age-related muscular atrophy. On the other hand, there is evidence that daily intake of EVOO, which contains high polyphenol levels, improves antioxidant status [21]. Therefore, it would be expected that the addition of EVOO would induce greater improvements. Reasons for the incapability of EVOO to improve skeletal muscle indexes are not promptly apparent, but it is clear that, in this study population, only EVOO without any dietary intervention could not improve said outcomes. In the present study, it is possible that the adoption of a healthy dietary pattern such as the DieTBra is enough to provide muscle tissue demand. It can be argued that the low content of processed and ultra-processed foods of the DieTBra already attenuated inflammation and oxidative stress with no further reduction by the EVOO; however, it was not in the scope of the present study to evaluate inflammatory markers. 

In the present study, we did not find increases in muscle mass between the three intervention groups. This finding could be explained due to the fact that our intervention, i.e., a hypocaloric diet, was not applied in conjunction with a physical activity intervention, such as resistance training [66]. Another important finding relates to the fact that EVOO on its own was not enough to improve muscle mass parameters in our participants. Stefan et al. [67] showed that a Mediterranean diet which includes high amount of olive oil consumption was associated with fat free mass. EVOO is known to play an important role in the control of apoptosis in the muscle through its antioxidant properties [68]. Therefore, a healthy diet accompanied by EVOO consumption, such as DieTBra and the Mediterranean Diet, is likely to promote an increase in the rates of protein synthesis and stimulated anabolic muscle response, which may also attenuate acute muscle loss [19].

Our main results confirm the importance of further research on dietary interventions in individuals with sarcopenic obesity from a preventive point of view [69]. Moreover, food-induced interventions are becoming increasingly relevant, given the current excessive consumption of ultra-processed foods, and because an inadequate diet facilitates a decrease in skeletal muscle mass [7,70,71].

The 12 week follow-up duration could be considered a limitation because it may have not been a long enough time to observe changes in muscle mass. However, we have shown some initial evidence on muscle strength and functionality despite the duration of our follow-up period. In fact, we believe that there was an underestimation. Thus, a longer follow-up would increase the magnitude of the results of the intervention of DieTBra and EVOO with further increases in muscle mass and improvements of other sarcopenia-related parameters in class II/III obesity. Another potential limitation could be attributed to the fact that ideally, we should have had a control group; i.e., one without intervention. However, the Ethics Committee was opposed to the idea of severely obese individuals being denied receiving any intervention. 

The nutritional interventions used in this study (EVOO and DieTBra) are based on real foods and not capsules/tablets that make it difficult for participants to comply during the long term. Real foods are a much better way to deliver nutritional interventions because they can be included and incorporated into the participant’s routine meals and do not cause any side effects. 

In randomized clinical trials, it is important to adopt statistical approaches that consider changes in variables during follow-up that may directly affect the outcomes. This was well illustrated in the present study: the body weight changes and the delta weight variable had direct effects on the analyzed outcomes, and thus, the statistical adjustments in the analysis of covariance were fundamental to indicating the effects of nutritional interventions.

## 5. Conclusions

The present findings showed that DieTBra improves both strength and muscle functionality through significant increases in handgrip strength and faster walking speed. DieTBra has also been associated with a reduction in total body fat in severely obese individuals. Extra virgin olive oil without DieTBra did not improve any of the outcomes investigated. These results support the importance of this nutritional intervention in the treatment of severely obese individuals by promoting gains in muscle function which is a big challenge in this group. Furthermore, our study makes an important contribution towards improvements of obesity class II/III treatment and sarcopenia prevention in obese individuals. 

## Figures and Tables

**Table 1:** Participants’ characteristics at baseline according to the three randomized intervention groups in adults with severe obesity, Brazil, 2016 (*n* = 111).

	Total *n* (%)	Olive oil (*n* = 34) *n* (%)	DieTBra (*n* = 38) *n* (%)	DieTBra + Olive Oil (*n* = 39) *n* (%)
Sex *				
Female	104 (93.7)	32 (94.1)	36 (94.7)	36 (92.3)
Male	7 (6.3)	2 (5.9)	2 (5.3)	3 (7.7)
Age *				
18–29	13 (11.7)	5 (14.7)	3 (7.9)	5 (12.8)
30–39	39 (35.1)	14 (41.2)	14 (36.8)	11 (28.2)
40–49	42 (37.9)	10 (29.4)	19 (50.0)	13 (33.3)
≥50	17 (15.3)	5 (14.7)	2 (5.3)	10 (25.7)
Skin color **				
White	34 (30.6)	12 (35.3)	9 (23.7)	13 (33.3)
Brown	60 (54.1)	17 (50.0)	24 (63.1)	19 (48.7)
Black	17 (15.3)	5 (14.7)	5 (13.2)	7 (18.0)
Marital status *				
Single	24 (21.6)	7 (20.6)	9 (23.7)	8 (20.5)
Married/Civil union	74 (66.7)	24 (70.6)	22 (57.9)	28 (71.8)
Widower/Divorced/Separated	13 (11.7)	3 (8.8)	7 (18.4)	3 (7.7)
Years of study *				
≤4	10 (9.0)	1 (2.9)	2 (5.3)	7 (17.9)
5 to 11	83 (74.8)	27 (79.4)	30 (78.9)	26 (66.7)
≥12	18 (16.2)	6 (17.7)	6 (15.8)	6 (15.4)
Economic Classification *				
Class A, B	28 (25.2)	12 (35.3)	7 (18.4)	9 (23.1)
Class C	65 (58.6)	15 (44.1)	24 (63.2)	26 (66.7)
Class D, E	18 (16.2)	7 (20.6)	7 (18.4)	4 (10.2)
Smoking *				
Non-Smokers	76 (68.5)	25 (73.5)	27 (71.0)	24 (61.5)
Ex-Smokers	28 (25.2)	7 (20.6)	9 (23.7)	12 (30.8)
Smokers	7 (6.3)	2 (5.9)	2 (5.3)	3 (7.7)
Binge drinking events (*n* = 107) **				
No	75 (70.1)	24 (70.6)	27 (73.0)	24 (64.9)
Yes	32 (29.9)	9 (29.4)	10 (27.0)	13 (35.1)
Aerobic physical activity (*n* = 104) *				
<150 min/week	101 (97.1)	31 (96.9)	35 (97.2)	35 (97.2)
≥150 min/week	3 (2.9)	1 (3.1)	1 (2.8)	1 (2.8)
BMI (kg/m^2^) *				
35–39	22 (19.8)	7 (20.6)	8 (21.0)	7 (18.0)
40–49	76 (68.5)	23 (67.6)	23 (60.5)	30 (76.9)
≥50	13 (11.7)	4 (11.8)	7 (18.5)	2 (5.1)
	**Mean ± SD**	**Mean ± SD**	**Mean ± SD**	**Mean ± SD**
ASMMI (kg/m^2^) ***	8.2 ± 1.3	8.45 ± 1.3	8.4 ± 1.2	7.8 ± 1.4
ASMM (kg) ***	21.0 ± 4.3	21.3 ± 4.3	21.5 ± 3.8	20.2 ± 4.7
ASMM/BMI ***	0.48 ± 0.11	0.50 ± 0.11	0.49 ± 0.10	0.47 ± 0.12
Total body fat (kg) ***	54.7 ± 8.0	52.8 ± 7.3	55.2 ± 8.3	55.9 ± 817
Percentage of body fat ***	51.7 ± 5.1	50.6 ± 5.0	51.5 ± 4.7	5.5 ± 52.6
Walking speed (m/s) ***	1.01 ± 0.18	1.02 ± 0.18	1.01 ± 0.17	1.02 ± 0.19
Handgrip strength (Kg) ***	22.9 ± 7.5	23.2 ± 9.6	23.6 ± 7.0	21.9 ± 5.7
Weight (kg) ***	110.9 ± 11.7	108.7 ± 10.9	112.2 ± 12.1	111.7 ± 11.9
Time of Sedentary behavior (min) (*n* = 93) ***	1162.8 ± 82.0	1159.1 ± 67.2	1154.1 ± 76.3	1173.8 ± 97.7
Total lean mass (Kg) ***	50.7 ± 9.1	49.7 ± 11.0	51.9 ± 7.6	50.4 ± 8.6
Fat free mass (Kg) ***	53.4 ± 8.0	53.49 ± 7.9	54.2 ± 7.4	52.6 ± 8.8

Note. SD = standard deviation; BMI = body mass index; ASMM = appendicular skeletal muscle mass index; ASMM = total appendicular skeletal muscle mass; ASMM/BMI = total appendicular skeletal muscle mass in relation to body mass index; * Fisher’s exact test; ** Pearson’s chi-square test; *** ANOVA.

**Table 2:** Analysis of outcomes between baseline and the end of follow-up according to intervention group adults with severe obesity.

	Olive Oil			DieTBra			DieTBra + Olive Oil		
	Mean ± SD Before (*n* = 34)	Mean ± SD After (*n* = 38)	*p* *	Mean ± SD Before (*n* = 38)	Mean ± SD After (*n* = 32)	*p* *	Mean ± SD Before (*n* = 39)	Mean ± SD After (*n* = 37)	*p* *
Weight (kg)	108.7 ± 10.9	108.5 ± 11.6	0.939	112.2 ± 12.1	111.7 ± 11.4	0.839	111.7 ± 11.9	109.0 ± 12.5	0.350
ASMMI (kg/m^2^)	8.4 ± 1.3	8.1 ± 1.3	0.243	8.4 ± 1.2	8.3 ± 1.4	0.720	7.8 ± 1.4	7.8 ± 1.2	0.913
ASMM (kg)	21.3 ± 4.3	20.2 ± 4.1	0.309	21.5 ± 3.8	21.4 ± 4.1	0.848	20.2 ± 4.7	20.0 ± 4.0	0.872
ASMM/BMI	0.50 ± 0.11	0.47 ± 0.10	0.265	0.49 ± 0.10	0.50 ± 0.11	0.925	0.47 ± 0.12	0.47 ± 0.10	0.882
Total body fat (kg)	52.8 ± 7.3	54.1 ± 8.5	0.509	55.2 ± 8.3	47.7 ± 20.6	0.041 ^†^	55.9 ± 8.1	52.4 ± 15.2	0.207
Percentage of body fat (%)	50.7 ± 5.0	51.9 ± 4.7	0.303	51.5 ± 4.7	51.4 ± 4.8	0.920	52.7 ± 5.5	52.7 ± 6.0	0.941
Walking speed (m/s)	1.02 ± 0.18	1.00 ± 0.18	0.683	1.01 ± 0.17	1.05 ± 0.21	0.359	1.02 ± 0.19	1.04 ± 0.14	0.632
Handgrip strength (kg)	23.2 ± 9.6	23.4 ± 6.2	0.905	23.6 ± 7.0	23.9 ± 7.0	0.854	21.9 ± 5.7	21.8 ± 5.9	0.934

Note. SD = standard deviation; BMI = body mass index; ASMMI = appendicular skeletal muscle mass index; ASMM = total appendicular skeletal muscle mass; ASMM/BMI = total appendicular skeletal muscle mass in relation to body mass index; HGS = handgrip strength; * unpaired Student’s *t*-test, ^†^ statistically significant difference.

**Table 3:** Analysis of outcome variables in the final stage of follow-up according to intervention groups, and analysis comparing groups two to two.

	Olive Oil (*n* = 38)	DieTBra (*n* = 32)	DieTBra + Olive Oil (*n* = 37)	*p* *	Olive Oil × DieTBra	Olive Oil × DieTBra + Olive Oil	DieTBra × DieTBra + Olive Oil
	Mean ± SD	Mean ± SD	Mean ± SD		*p* **	*p* **	*p* **
Weight (kg)	108.5 ± 11.6	111.37 ± 11.4	109.0 ± 12.5	0.592	0.332	0.850	0.422
ASMMI (kg/m^2^)	8.1 ± 1.3	8.3 ± 1.4	7.8 ± 1.2	0.249	0.425	0.432	0.100
ASMM (kg)	20.2 ± 4.1	21.4 ± 4.1	20.0 ± 4.0	0.361	0.281	0.868	0.181
ASMM/BMI	0.47 ± 0.10	0.50 ± 0.11	0.47 ± 0.10	0.486	0.288	0.862	0.334
Total body fat (kg)	54.1 ± 8.5	55.2 ± 8.3	55.3 ± 8.9	0.852	0.637	0.606	0.963
Percentage of body fat (%)	51.9 ± 4.7	51.4 ± 4.8	52.7 ± 6.0	0.575	0.672	0.561	0.319
Walking speed (m/s)	1.00 ± 0.18	1.06 ± 0.20	1.04 ± 0.14	0.375	0.215	0.319	0.572
HGS (kg)	23.4 ± 6.2	23.9 ± 7.1	21.8 ± 5.9	0.368	0.770	0.296	0.186

Note. SD = standard deviation BMI = body mass index; ASMMI = appendicular skeletal muscle mass index; ASMM = total appendicular skeletal muscle mass; ASMM/BMI = total appendicular skeletal muscle mass in relation to body mass index; HGS = handgrip strength; * ANOVA; ** Student’s *t*-test.

**Table 4:** Delta (effectiveness) of outcome variables according to intervention groups and paired comparison.

	Olive Oil (*n* = 38)	DieTBra (*n* = 32)	DieTBra + Olive Oil (*n* = 37)	*p* *	Olive Oil × DieTBra	Olive Oil × DieTBra + Olive Oil	DieTBra × DieTBra + Olive Oil
	Mean ± SD	Mean ± SD	Mean ± SD		*p* **	*p* **	*p* **
∆ Weight (kg)	0.98 ± 2.26	−1.38 ± 3.41	−1.29 ± 2.72	0.002 ^†^	0.003 ^†^	0.001 ^†^	0.904
∆ ASMMI (kg/m^2^)	−0.42 ± 0.96	−0.22 ± 1.04	−0.15 ± 1.08	0.553	0.441	0.285	0.765
∆ ASMM (kg)	−1.05 ± 2.29	−0.57 ± 2.68	−0.40 ± 2.80	0.600	0.466	0.318	0.789
∆ ASMM/BMI	−0.03 ± 0.05	−0.01 ± 0.06	−0.01 ± 0.06	0.178	0.125	0.073	0.810
∆ Total body fat (kg)	−1.16 ± 2.72	−0.34 ± 3.46	−0.45 ± 2.90	0.077	0.070	0.026 ^†^	0.881
∆ Percentage of body fat (%)	0.64 ± 2.43	0.27 ± 2.59	0.13 ± 2.25	0.690	0.575	0.380	0.801
∆ Walking speed (m/s)	−0.01 ± 0.13	0.04 ± 0.15	0.01 ± 0.15	0.283	0.108	0.567	0.302
∆ Handgrip strength (Kg)	1.14 ± 5.64	0.31 ± 4.49	−0.05 ± 4.09	0.593	0.528	0.325	0.724

Note. SD = standard deviation; BMI = body mass index; ASMMI = appendicular skeletal muscle mass index; ASMM = total appendicular skeletal muscle mass; ASMM/BMI = total appendicular skeletal muscle mass in relation to body mass index; ∆ = delta, difference between end of follow-up and baseline; ANOVA Test *; ** Student’s *t*-test; ^†^ statistically significant difference.

**Table 5:** Analysis of covariance for delta of outcome variables * with delta weight and delta time of sedentary behavior in intervention groups.

	Olive Oil (*n* = 38)	DieTBra (*n* = 32)	DieTBra + Olive Oil (*n* = 37)
	*p*	*p*	*P*
Total body fat (kg)	-	-	0.001 ^¥^
∆ Total body fat (kg)	-	0.016 ^†^	0.004 ^†^
∆ Walking speed (m/s)	-	0.042 ^†^	-
∆ Handgrip strength (Kg)	-	0.044 ^†^	-

Note. ∆ = delta, difference between end of follow-up and baseline * variables that met ANCOVA requirements. ^†^ statistically significant difference adjusted by delta weight; ^¥^ statistically significant difference adjusted by delta time of sedentary behavior.
